# Interventions to promote early child development in the Middle East and North Africa Arab countries: protocol for a scoping review

**DOI:** 10.1080/16549716.2026.2720377

**Published:** 2026-08-27

**Authors:** Lama Charafeddine, Mona Nabulsi, Maya Al Farran, Catherine Hamzeh, Ola El Zein, Alya Alsager, Sham Aldos, Michelle Shah, Wafaie Fawzi, Aisha K. Yousafzai

**Affiliations:** a Department of Pediatrics and Adolescent Medicine, American University of Beirut, Beirut, Lebanon; b Department of Medicine and Health Sciences, American University of Beirut, Beirut, Lebanon; c Clinical Research Institute, Faculty of Medicine, American University of Beirut, Beirut, Lebanon; d Department of Global Health and Population, Harvard T.H. Chan School of Public Health, Boston, USA

**Keywords:** Parenting, cognition, language development, motor skills, emotions

## Abstract

The Middle East and North Africa Arab countries face several economic, political, and social challenges that negatively impact early child physical, cognitive, and emotional development. This scoping review aims to summarize the landscape of interventions designed to promote early child development in children under 5 years of age in the Middle East and North Africa Arab countries, highlighting knowledge gaps and areas for future research. We searched Embase, PsycINFO, MEDLINE OVID, and Scopus databases from conception for randomized and non-randomized trials reporting early child development interventions in children below 5 years of age in these countries, published in English, French, or Arabic. We also searched the grey literature and reference lists of relevant peer-reviewed studies and reviews identified in the screening process. Two independent reviewers screened titles and abstracts of retrieved papers for inclusion with discrepancies resolved by discussion among three reviewers, and similarly for reviewing the full text of papers that passed the title/abstract screening phase. We extracted and charted data on study design, participant characteristics of the intervention and comparison groups, study setting, intervention details, primary and secondary outcomes, and key findings using a pre-piloted standardized data extraction form. Data extraction and charting were done independently and in duplicate. The findings were summarized as a descriptive numerical summary of key study characteristics, as well as a thematic narrative synthesis of the intervention types, target populations, and outcomes. The protocol was prospectively registered in Open Science Framework before searching, study selection, and data extraction commenced.

## Paper Context

- **Main findings:** The Middle East and North Africa Arab countries face several economic, political, and social challenges that can adversely affect early child development
- **Added knowledge:** There is a paucity of the literature on early child development interventions from this region.
- **Global health impact for policy and action:** This scoping review will highlight critical knowledge gaps pertaining to early child development interventions to inform future research and guide policy initiatives in Arab countries.

## Background

The first 5 years of life represent a sensitive period for a child’s development, laying the foundation for their physical, cognitive, social, and emotional well-being. Research has proven that significant brain development occurs during pregnancy and the early years of life, shaped by a complex interplay of genetic factors and early experiences [1]. These experiences are vital, influencing both immediate development and long-term social and emotional well-being in adolescence and adulthood [1]. Supportive home learning environments, characterized by parental involvement, contribute to positive cognitive, language, and socio-emotional development, and enhancing parental knowledge and practices further promotes children’s potential [2]. Responsive caregiving and positive caregiver–child relationships stimulate brain development through mutual engagement and enhancing abilities like empathy and self-control [1].

In low- and middle-income countries, child poverty presents a complex and multidimensional challenge that may weaken children’s physical, cognitive, and emotional development [2]. Economic instability, political unrest, and the ongoing crises and wars in the Middle East and North Africa (MENA) Arab region increase risks of impaired cognitive abilities, delayed language skills, and increased emotional and behavioral issues, ultimately compromising children’s future social prospects [2].

Two recent global systematic reviews showed that parenting interventions, particularly those delivered during the first 3 years of life, can improve cognitive, language, motor, and socioemotional development in children, and enhance caregivers’ parenting practices [3,4]. These interventions had a stronger impact in low- and middle-income countries compared to high-income countries (HICs), emphasizing the potential for greater benefits in resource-limited regions like the MENA countries [3]. Both reviews highlighted the need for focused research and interventions in the MENA region to address these critical gaps [3,4]. A recent national survey from Lebanon on the status of nutrition and early child development showed that malnutrition, particularly stunting, dramatically increases the risk of developmental deficits [5]. This highlights the severe cognitive capital loss associated with malnutrition, especially in countries where food insecurity remains a significant challenge [5].

### Objectives/scoping review questions

The scoping review’s objective is to systematically map the literature and summarize the evidence available on interventions aimed at promoting early child development (ECD) in children under 5 years of age in the MENA Arab countries and answer the following questions:

1. What are the types of interventions implemented, their implementation features, and the target populations?
2. How effective are these interventions in promoting early child development?
3. Are the interventions culturally aligned, accessible, and are they cost effective?
4. What are the existing knowledge gaps and areas for potential action and future research in this region?

## Methods

### Protocol and registration

The protocol of this scoping review was developed in accordance with the Preferred Reporting Items for Systematic Reviews and Meta-Analyses Extension for Scoping Reviews (PRISMA-Sc) Checklist [6] (Supplementary File S1). The protocol was prospectively registered on 12 November 2024 in Open Science Framework (OSF): https://archive.org/details/osf-registrations-ehuqx-v1 before searching databases, study selection, and data extraction commenced. Publication of the protocol occurred after data collection owing to delays in the publication process.

### Eligibility criteria

The review questions and eligibility criteria were structured using the Population-Concept-Context (PCC) framework [7]. Type of studies: We included randomized and non-randomized trials conducted in the MENA Arab countries on children below 5 years of age. All other study designs were excluded. Restricting eligibility to intervention trials that have a comparator was because they are the best study designs to investigate the effectiveness of interventions and hence can guide clinical practice, policy makers, and future research. The MENA Arab countries of interest include the 22 countries of the Arab League: Algeria, Bahrain, Comoros, Djibouti, Egypt, Iraq, Jordan, Kuwait, Lebanon, Libya, Mauritania, Morocco, Oman, Qatar, Kingdom of Saudi Arabia, Somalia, Sudan, Syria, Tunisia, the United Arab Emirates, Palestine (West Bank & Gaza), and Yemen. Multi-country studies were included if they presented data on at least one of the Arab countries. This inclusive approach allows for a comprehensive understanding of the different types of ECD interventions implemented in these countries, their effectiveness, and their contextual factors. Eligible studies included both peer-reviewed articles and grey literature published in English, French, or Arabic, without restrictions on publication dates.

*Population/Participants*: This scoping review focused on children below 5 years of age and their caregivers in the MENA Arab countries, irrespective of their health or social status. Hence, it included healthy children as well as children with developmental disorders or disabilities, or who were refugees or displaced from their homes. The focus on the first 5 years of life is because it is a sensitive developmental period where biological and/or psychosocial interventions can significantly influence long-term brain development and psychosocial outcomes [8].

Concept: The concept of interest was any evaluated intervention intended to promote early child development. Interventions could target children, caregivers, caregiver–child interactions, or early child environments and could include, but were not limited to, parenting guidance and support, children’s media, clinical or therapeutic services, and early childhood care and education services, regardless of their scale or complexity. We charted intervention aims, content, delivery modalities, implementation features, contextual adaptations, and reported child- and caregiver-level outcomes. As highlighted by Phillips and Shonkoff [9], early childhood intervention is more appropriately understood as a broad concept than as a single type of program, reflecting the diversity of relevant interventions.

Context: The context included any home, community, healthcare, humanitarian, or early childhood care and education setting within the 22 MENA Arab countries listed above. Multi-country studies were eligible when they reported disaggregated data for at least one eligible Arab country.

Comparators: We included studies that compared an ECD intervention to standard of care, no intervention, or another ECD intervention.

Outcomes: Our primary outcomes included any early child developmental outcome like attachment, cognitive development, language (expressive, receptive), motor skills (fine, gross), social-emotional development, and behaviour. Secondary outcomes included parenting knowledge and practices, caregiver–child interactions, stimulation/early learning practices, parent/caregiver mental health, violence against children, responsive care and feeding practices, home environment, early care and education environment, and caregiver/teacher knowledge and practices. Given the long list of secondary outcomes, based on the final data extraction, these were clustered into themes.

### Search strategy

Comprehensive literature search strategies using controlled vocabularies and relevant text words related to ECD interventions targeting children under 5 years of age and their caregivers in the MENA Arab countries were developed. We searched MEDLINE OVID, Embase, APA PsycINFO, and Scopus, with no restrictions on publication date or language. We also searched the grey literature by including resources such as ClinicalTrials.gov and the WHO platform for clinical trials to identify ongoing or recently completed trials, regional platforms like Manhal (منهل) and Maarif (معارف) to gather non-peer-reviewed literature, and organizations involved in ECD like United Nations Children’s Fund, World Health Organization, the World Bank, the Arab Resource Network, the Early Child Development Action Network, New York University Ties, Save the Children, the International Rescue Committee, World Vision, Plan International, the Lego Foundation, the Aga Khan Foundation, and the Bernard van Leer Foundation. Additionally, we reviewed the reference lists of included studies and relevant reviews to further identify studies not captured by the electronic database search. This comprehensive approach ensured a thorough examination of the current landscape of ECD interventions in the MENA Arab countries.

The search strategy was developed by the director of evidence synthesis unit (OZ) at the Clinical Research Institute, American University of Beirut who is an information specialist expert in evidence synthesis. OZ first developed a MEDLINE search strategy (Table 1) that was adapted to the syntax and subject headings of the other databases. The project team that includes experts in ECD and systematic reviews provided input on the search strategy. The search in Manhal and Maarif used Arabic keywords of the same search concepts. The search was done by two independent Arabic speaking researchers supported by the Arabic speaking principal investigators and information specialist. The initial database searches were conducted in December 2024. Alerts were created in each database to continue receiving any newly published article relevant to our search during the screening process. Newly published studies captured through these alerts were reviewed for eligibility before final inclusion. For the search, we used controlled vocabularies and text words related to the following concepts: early child development, children under the age of 5 years, and the 22 MENA Arab countries, combined with Boolean operators as shown in Table 1.

**Table 1.** MEDLINE search strategy.

No.	Concept	MEDLINE search terms
1	ECD	communication/ or narration/ or child rearing/ or motivation/ or empowerment/ or family relations/ or maternal behavior/ or parent-child relations/ or father-child relations/ or mother-child relations/ or parenting/ or paternal behavior/ or parents/ or fathers/ or adolescent fathers/ or mothers/ or single parent/ or surrogate mothers/
2	ECD	social support/ or family support/ or psychosocial support systems/ or education/ or mentoring/ or teaching/
3	ECD	((parent* or maternal or paternal or father* or mother* or grandfather* or grandmother* or caregiv* or carer* or (care adj giv*) or grandparent* or homehelp* or ((home? or house? or domestic) adj (help* or keep* or cleaner? or chores or employee? or worker? or labor? or serv?nt?)) or nann* or maid? or housemaid? or chambermaid? or housewi?e? or housekeeper* or maidservant? or handmaiden? or skivv* or charwom?n or char-wom?n or attendant or menial? or baby-sitter? or ((baby or babies) adj (watch* or care* or sitter?)) or babysitter?) adj3 (child* or toddler* or kid or kids or p?ediatr* or minor? or infan* or neonat* or newborn* or (new adj born*) or (pre adj schooler*) or preschooler* or baby or babies) adj3 (relation* or rear* or nurtur*)).mp.
4	ECD	Caregivers/ or Housekeeping/
5	ECD	((parent* or maternal or paternal or father* or mother* or grandfather* or grandmother* or caregiv* or carer* or (care adj giv*) or grandparent* or homehelp* or ((home? or house? or domestic) adj (help* or keep* or cleaner? or chores or employee? or worker? or labor? or serv?nt?)) or nann* or maid? or housemaid? or chambermaid? or housewi?e? or housekeeper* or maidservant? or handmaiden? or skivv* or charwom?n or char-wom?n or attendant or menial? or baby-sitter? or ((baby or babies) adj (watch* or care* or sitter?)) or babysitter?) adj4 (educat* or coach* or mentor* or support* or train* or workshop* or session* or counsel* or advice? or advis* or communicat* or narrat* or motivat* or empower* or behav* or act or acts or acting)).mp.
6	Total ECD	1 or 2 or 3 or 4 or 5
7	Children: 0–5 years old	exp infant/ or exp child/ or minors/ or child, abandoned/ or child, adopted/ or child, exceptional/ or child, gifted/ or ‘child of impaired parents’/ or child, foster/ or child, orphaned/ or child, unwanted/ or child day care centers/ or nurseries, infant/
8	Children: 0–5 years old	(child or children or childhood or toddler* or kid or kids or p?ediatr* or minor? or infan* or neonat* or newborn* or (new adj born*) or (pre adj schooler*) or preschooler* or baby or babies or premature* or pre-mature* or preterm or pre-term or premie* or post-nat* or postnat* or preschoolchild* or preschoolboy? or preschoolgirl? or pre-schoolchild* or pre-schoolboy? or pre-schoolgirl? or minor?).mp.
9	Children: 0–5 years old	((preschool* or (pre adj school*) or nurser* or daycare* or (day adj care*) or creche* or garderie* or kindergarten* or (kinder adj garten*) or (primary adj school*)) adj3 (boy? or girl? or student* or child or children or infan*)).mp.
10	Total Children: 0–5 years old	7 or 8 or 9
11	Arab Region	algeria/ or bahrain/ or comoros/ or djibouti/ or egypt/ or iraq/ or jordan/ or kuwait/ or lebanon/ or libya/ or mauritania/ or morocco/ or oman/ or qatar/ or saudi arabia/ or somalia/ or sudan/ or south sudan/ or syria/ or tunisia/ or united arab emirates/ or yemen/ or Arabs/ or middle east/ or africa, northern/ or exp ‘middle eastern and north africans’/
12	Arab Region	(Algeri* or Bahr?in* or Bahr?yn* or Manama or Comoro or Comoros or Moroni or Djibouti* or Egypt* or Cairo or Irak* or Iraq* or Bag?dad* or Jordan* or Amman or k?w?it* or L?ban* or Be?rut* or Ba?rut* or Liby* or Libia* or tripoli or tarab?l?s or Ma?ritani* or Mo?ritani* or Mu?ritani* or No?ak?hot* or m?roc* or Ifni or R?bat or marakesh or Oman* or Mus?at* or Palestin* or G?az?a* or J?ru?alem or (Occupied adj2 territor*) or (West* adj2 bank) or Q?atar* or Katar* or Doha or Saudi* or KSA or Riyad* or jedda* or So?mal* or m?g?adish* or M?q?adish* or M?k?adish* or S?udan* or Kharto?m or Khart?um or Syria* or Syrie* or Tunis* or Emirat* or (Ab?u adj2 D?abi) or UAE or EAU or Dubai or Sharja? or Ajman or ((Um* or Om*) adj2 (Kiwe?n or quwe?n or Quw?in)) or Fuja?ra? or Fuje?ra? or (Ras adj2 Khayma*) or Yaman or Yemen or Yamani or Yemeni or Sanaa or Sana’a or Aden or Gulf or (North* adj3 Africa*) or Mag?r?b* or Sahara or (Middle adj3 East*) or (Near adj2 East*) or (East* adj2 Mediterranean) or (bedouin* adj2 (countr* or state*)) or Arab or Arabs or Arabia or Levant or MENA or Orient).mp.
13	Total Arab Region	11 or 12
14	Total	6 and 10 and 13

### Data management

All literature search results were exported to Covidence (Veritas Health Innovation, Melbourne, Australia. Available at www.covidence.org) for storing the search results, removing duplicates, and managing the entire screening process. Titles and abstracts of all retrieved papers were screened independently by two authors using predefined guiding questions that operationalized the Population-Concept-Context eligibility criteria described above, together with the prespecified study-design requirements. Any study that received conflicting decisions was flagged for further discussion by three authors (LC, MN, AKY) to resolve conflicts and reach consensus. For the full-text review, papers were independently assessed by two reviewers for inclusion/exclusion in the scoping review. Disagreements were similarly resolved by discussion among the same three authors to reach consensus. At the end of the full-text screening, the selection process was summarized in a PRISMA flow diagram, detailing the total number of included and excluded studies, as well as the reasons for exclusion.

### Data charting

We developed and tested a data charting form in Excel prior to data extraction. Three authors independently piloted the form on three papers to further refine it. After validation and approval of the form by all investigators, two reviewers extracted data from each study independently. Any discrepancies in data extraction were resolved through discussion among three reviewers (LC, MN, AKY). Study authors were contacted by email in case of missing or unclear data.

### Data items

We extracted and charted the following information from each included study:

- Study identification: Title, authors, journal name/data source, date of publication, country of study, type of study design (randomized/quasi-randomized), funding source and role of funding, and conflict of interest.
- Study purpose and methodology: Aim, study design, setting details (urban/rural), ethics approval.
- Study details:
  - Population: country, age range of children, child characteristics (healthy, impoverished, refugee, malnourished, disability, preterm), caregiver targeted (mother, father, andteacher), caregiver characteristics (first-time parent, depressed), andnumber of participants (intervention and control).
  - Intervention: description of the intervention (parenting program, play-based learning, etc.), delivery (individual, group, in-person, digital, etc.), setting (home, clinic, school, community, etc.), person delivering intervention (nurse, teacher, health care worker, community worker), context specific (intervention designed/adapted to context), dosage (duration/frequency of sessions), cost (if available), fidelity of implementation, sustainability or scaling, implementation factors (barriers and facilitators).
  - Comparator: description of control group (if different from intervention group), description of the comparator intervention (no intervention, standard of care, another ECD intervention).
  - Outcomes: primary outcomes (socio-emotional, cognitive, language, motor), secondary outcomes, problem-solving, parenting/caregiver, caregiver–child interaction, stimulation/early learning practices, parent/caregiver mental health, parent/caregiver education, and violence against children.
  - Outcome measure reliability/validity: measures adapted/local, information on reliability, and information on validity.
  - Cultural and contextual alignment were charted according to whether the intervention was locally developed or adapted and any reported processes or modifications supporting local relevance, including translation, formative research or needs assessment, stakeholder consultation, piloting, and adaptation of intervention content or delivery to local sociocultural norms, resource conditions, institutional arrangements, service structures, or family routines. Accessibility was charted through the reported delivery setting and modality, delivery personnel, adaptations for displaced or resource-constrained populations, and available information on implementation barriers and facilitators, reach, engagement, retention, and attrition. Cost and resource information included reported programme or per-participant costs, staffing, training, materials, technology, participant support, and any formal economic evaluation. These dimensions were summarized descriptively as reported in the studies.
  - Findings relevant to each outcome.
  - Additional comments/observations.

### Collating, summarizing, and reporting results

The number of records identified, screened, included, and excluded at each stage of the review was summarized in a PRISMA flow diagram. We adopted a descriptive-analytical approach to summarize the studies that met our inclusion criteria. We retained studies with unreported or incomplete data in the narrative synthesis noting their limitations for the purpose of thorough mapping of the entire evidence landscape. We did not assess the risk of bias in the included papers as this is not within the objectives of a scoping review, in contrast to a systematic review where assessment of the rigor of included studies is an essential step in the conduct of systematic reviews. The analysis included two complementary components. First, we produced a descriptive numerical summary of the included studies to describe publication trends, geographic distribution, study designs, intervention characteristics, and reported outcomes; these findings were presented in tables and figures. Second, we conducted a structured narrative thematic synthesis during data charting. Extracted data were compared across studies, and categories were developed iteratively by the review team. Intervention types were developed inductively from each intervention’s stated programmatic objective and grouped as parenting guidance and support, clinical or therapeutic services, or early childhood care and education services. Target child populations were classified as children with developmental disabilities, children with other health or nutritional vulnerabilities, or typically developing children, with additional consideration of contextual vulnerabilities such as conflict exposure, displacement, and poverty. Child- and caregiver-level outcomes were grouped into broader domains to facilitate comparison across studies.

## Discussion

To our knowledge, this is the first scoping review to map the landscape of interventions aimed at promoting early child development in children under 5 years of age in the MENA Arab countries. It will highlight critical knowledge gaps that impede effective policy and practice in ECD in the region. The findings will contribute to the advancement of the field by offering a deeper understanding of available interventions and their outcomes, thereby informing future research and guiding policy initiatives that support early child development across the MENA region. Potential limitations of this review may be the paucity of the literature on early childhood interventions designed to promote child development in the MENA Arab countries, and/or the lack of high-quality randomized trials.

Early childhood interventions are diverse in terms of their scope, format, sector, implementation, and target groups. These interventions can range from broad health promotion and disease prevention initiatives to specialized programs addressing developmental disabilities, family violence, and mental health challenges. Consequently, the targeted populations can vary significantly, leading to a heterogeneous group with diverse needs and circumstances. Interventions may adopt various service formats, including center-based and home-based models, and may incorporate differing philosophies that blend child-focused and family-focused approaches. Additionally, there could be considerable variability in staffing configurations; interventions can be delivered by highly trained professionals, such as educators and therapists, or by community workers with varying levels of formal education and training. Hence, we will include all types of interventions that target our research question regardless of their size or scope, hoping to explore and analyze the full spectrum of interventions being implemented in the MENA Arab countries and ensuring a comprehensive understanding of the current landscape. By synthesizing a wide array of evidence, this scoping review will inform future research and policy initiatives in the field of ECD in the MENA region.

## Supplementary Material

Supplementary File 1.docx

## Patient and public involvement

Patients were not involved in this scoping review.
